# Supplementary material for: Severe cardiac and skeletal manifestations in DMD-edited microminipigs: an advanced surrogate for Duchenne muscular dystrophy
Source: Commun Biol. 2024 May 3;7:523. doi: 10.1038/s42003-024-06222-5 (PMC11068776; doi:10.1038/s42003-024-06222-5)
Supplement: Supplementary file 2 — Supplementary Information [file 42003_2024_6222_MOESM2_ESM.pdf]

Supplementary Table 1: Microminipigs used in the study

Subject	Genotype	Date of birth	Date of death	Age (months)	Weight (kg)	Cause of death
F2-03	DMD	2019/1/10	2021/6/25	29.9	17	Experiment
F3-02	DMD	2022/5/8	2023/5/24	12.7	19	Experiment
F3-03	DMD	2022/5/8	2023/5/24	12.7	19	Experiment
F2-05	DMD	2020/1/24	2020/7/24	6.1	9.3	Natural*
F2-07	DMD	2021/4/26	2021/8/31	4.2	6.6	Natural*
F2-06	DMD	2020/8/14	2020/12/18	4.2	5.1	Natural*
F3-04	DMD	2022/11/15	2023/2/25	3.4	5.7	Natural*
F2-04	DMD	2019/1/10	2019/4/22	3.4	5.4	Natural*
F2-12	DMD	2023/1/30	2023/4/21	2.7	5.8	Natural*
F2-10	DMD	2022/7/1	2022/8/30	2	3.2	Experiment
F2-11	DMD	2022/10/29	2023/4/27	6	8	Experiment
F2-13	DMD	2023/2/11	-	-	-	-
F2-14	DMD	2023/2/11	-	-	-	-
WT-01	WT	2019/1/21	2021/6/25	29.5	24	Experiment
WT-03	WT	2022/5/8	2023/5/24	12.7	20	Experiment
WT-04	WT	2022/6/19	2023/5/24	11.3	16	Experiment
WT-02	WT	2020/8/14	2021/3/5	6.8	9.3	Experiment
WT-05	WT	2022/11/23	-	-	-	-
WT-06	WT	2022/11/23	-	-	-	-
WT-07	WT	2022/11/15	2023/3/8	3.8	6	Experiment
WT-08	WT	2023/1/30	2023/4/27	2.9	6.3	Experiment
WT-09	WT	2022/7/1	2022/8/30	2	4.4	Experiment
WT-10	WT	2022/10/29	2023/5/2	6.1	9.1	Experiment

An asterisk in the Cause of death column indicates sudden death.

Supplementary Table 2: Pigs used for generation of DMD-edited microminipigs

Strain	subject	Genotype	sex	Date of birth	Date of Exam.	Age(month)	Purpose of use
Duroc	15-0192	WT	female	2015/6/15	2016/5/25	11.5	surrogate mother sow for recipient.
Duroc	15-0210	WT	female	2015/6/16	2016/5/25	11.5	surrogate mother sow for recipient.
LargeWhite	15-0491	WT	female	2015/10/19	2016/5/25	7.3	surrogate mother sow for recipient.
MMP	15-4151	WT	female	2015/7/24	2016/5/25	10.2	embryo donation for generation of F0
MMP	15-4166	WT	female	2015/8/8	2016/5/25	9.7	embryo donation for generation of F0
MMP	15-4168	WT	female	2015/8/8	2016/5/25	9.7	embryo donation for generation of F0
MMP	15-4182	WT	female	2015/8/24	2016/5/25	9.2	embryo donation for generation of F0
MMP	15-4188	WT	female	2015/9/6	2016/5/25	8.7	embryo donation for generation of F0
MMP	15-4139	WT	male	2015/7/10	2016/5/25	10.7	mateing with embryo donor female.
MMP	15-4177	WT	male	2015/8/13	2016/5/25	9.5	mateing with embryo donor female.
MMP	13-3050	WT	male	2013/10/29	2016/5/25	31.3	mateing with embryo donor female.
MMP	15-4238	WT	female	2016/1/12			mateing with F0-4 for prodction of F1
MMP	16-4321	WT	female	2016/7/14			mateing with F0-4 for prodction of F1
MMP	16-4329	WT	female	2016/7/27			mateing with F0-4 for prodction of F1
MMP	16-4330	WT	female	2016/7/27			mateing with F0-4 for prodction of F1
MMP	F0-04	mosaic	male	2016/9/12			mating with wild-type MMPs

MMP= microminipig, F0= Founder

Supplementary Table 3. List of putative off-target sites homologous to sgRNA

	Sequence of off-target	Mismatch	Chromosome	Location	
on-target	GGGCAGACAGTTTCTTCCAC CGG	0	6	142243354	142243376
mm2_1	GGGCAGACAGTATCTGCCAC AGG	2	6	142243354	142243376
mm3_1	GGACAGACAGTTAGTTCCAC TGG	3	15	81895047	81895069
mm3_2	GGAGAGACAGTCTCTTCCAC TGG	3	Y	4327986	4328008
mm3_3	GGAGAGACAGTCTCTTCCAC TGG	3	X	5784995	5785017
mm3_4	GGACAGAGAGCTTCTTCCAC GGG	3	unknown	45010	45032
mm3_5	GTGCAGAAAGCTTCTTCCAC TGG	3	1	271880001	271880023
mm3_6	GGCCAGATATTTTCTTCCAC AGG	3	13	26558116	26558138
mm3_7	GGGCAGACAGCTTCATCAAC AGG	3	15	13715296	13715318
mm3_8	GGGAAGTCAGTTTCTTACAC TGG	3	10	45572841	45572863
mm3_9	GGGAAGGCAGTTTCCTCCAC TGG	3	17	12856693	12856715
mm3_10	TGGCAGTCAGTTTCTGCCAC TGG	3	10	27587084	27587106
mm3_11	GGGCAGACAGATGCTTCTAC AGG	3	1	31669919	31669941
mm3_12	GGCCCGAGAGTTTCTTCCAC GGG	3	7	53899311	53899333
mm3_13	GCAAAGACAGTTTCTTCCAC AAG	3	1	52685167	52685189
mm3_14	GGCCAGACAGATTCTGCCAC AGG	3	3	114626442	114626464
mm3_15	TGGCAGACAGTTTCTTTCCC AGG	3	2	39914866	39914888
mm3_16	GGGCAGACCTTTTCTTCCCC GGG	3	3	30747829	30747851
mm3_17	GGGCAGACAGTTTCTTTCTT AGG	3	7	38936134	38936156
mm3_18	GGTCAGACAGTTTCATCCCC TGG	3	14	49369619	49369641
mm3_19	GGGCAGACAGTCTCTTCCCT GGG	3	8	16654511	16654533
mm3_20	TGACACACAGTTTCTTCCAC TGA	3	13	29413564	29413586
mm3_21	GGGCAGACAGCTCCTTCCAG GGG	3	10	4576094	4576116
mm3_22	GGGCAGAAAGTTCCTTCCAG TGG	3	8	3395830	3395852
mm3_23	GGCTTGACAGTTTCTTCCAC TGA	3	6	116329416	116329438
mm3_24	GGGCAGACATTTTGTCCCAC AGG	3	4	80481399	80481421
mm3_25	GTGCAGACAGTTTGTCCCAC GGG	3	3	59360438	59360460

Supplementary Table 4. Sequences of primers used for PCR amplification of the off-target sites

Name	PCR	Forward	Reverse
mm2_1	1st	5'-CCCAAAAGTCCCTCAGACAG-3'	5'-GCCTTGGGCAAAACACTTAAC-3'
	2nd	5'-CCCTCAGACAGACTTCCCCT-3'	5'-GGGCAAAACACTTAACTTCT-3'
mm3_1	1st	5'-GGAGAGGCTTTTGCACTGAG-3'	5'-CCACTCAGTGTTGGTCGTGT-3'
	2nd	5'-GGCTTTTGCACTGAGAAAGC-3'	5'-CAGTGTTGGTCGTGTCTGCC-3'
mm3_2	1st	5'-CACCCAGGAGAGGCTTTTGC-3'	5'-CCACTCAGTGTTGGTCGTGT-3'
	2nd	5'-GGAGAGGCTTTTGCACTGAG-3'	5'-GTGTTGGTCGTGTCTGCCAG-3'
mm3_3	1st	5'-AGTTGAAACTCCCGCATCAC-3'	5'-CCCGTCTAACTGGCATTCTC-3'
	2nd	5'-CTCCCGCATCACCTCTCTGA-3'	5'-CTAACTGGCATTCTCTGAGA-3'
mm3_4	1st	5'-TGTAAGCCTCTGCACACGAC-3'	5'-TTTCCGTTTTTCAAGGTGGT-3'
	2nd	5'-GCCTCTGCACACGACAAAAC-3'	5'-GTTTTTCAAGGTGGTGCCCT-3'
mm3_5	1st	5'-ACTTGGGTACTGGCATCCTG-3'	5'-GCCTCCTTCCTACTGCCTCT-3'
	2nd	5'-GGTACTGGCATCCTGAGCCT-3'	5'-CCTACTGCCTCTCTGTGAAG-3'
mm3-6	1st	5'-AAACCTCATCGGAACACTGG-3'	5'-TCTTCTGTCAGTGCCCTCCT-3’
	2nd	5'-CTCATCGGAACACTGGAGGG-3'	5'-GTCAGTGCCCTCCTTGGCCA-3'
mm3_7	1st	5'-GAGAGACAGCATCCAGCTCA-3'	5'-AAAGGAAAGGAACGGAGGAA-3'
	2nd	5'-GACAGCATCCAGCTCATAGT-3'	5'-AGGAACGGAGGAAGGAAGGA-3'
mm2_8	1st	5'-AAATTGCCTCTTCATGCCTTA-3'	5'-GGTTTCAACCAGCTAATTCACC-3'
	2nd	5'-GCCTCTTCATGCCTTAAGGA-3'	5'-CCAGCTAATTCACCACCTAC-3'
mm3_9	1st	5'-GAACAGCAATGCCATTCTCA-3'	5'-GCATTCCTTCTGGTGGAAAA-3'
	2nd	5'-GCAATGCCATTCTCAGGAGC-3'	5'-CCTTCTGGTGGAAAACTAGC-3'
mm3_10	1st	5'-ATCACCAAGGGGACTCTTCC-3'	5'-TCCTTCATGCTTTTCCATCC-3'
	2nd	5'-CAAGGGGACTCTTCCTATTT-3'	5'-CATGCTTTTCCATCCTTATA-3'
mm3_11	1st	5'-GAGACTCCCAGCTGGTTCTG-3'	5'-GCCCGGACTTCTTAGTTCCT-3'
	2nd	5'-CCCAGCTGGTTCTGAAAGCC-3'	5'-GACTTCTTAGTTCCTCCAGG-3'
mm3_12	1st	5'-GTCAATATGCACCGCTTCTG-3'	5'-TCTCTCAACTGTGCCCTCCT-3'
	2nd	5'-TGCACCGCTTCTGTTGATAA-3'	5'-CAACTGTGCCCTCCTGGCTT-3'
mm3_13	1st	5'-TGCTGTGGCTATGGTGTAGC-3'	5'-ACTGACTGGCTGGAGCACTT-3'
	2nd	5'-TGGCTATGGTGTAGCCTGGA-3'	5'-CTGGCTGGAGCACTTTACAC-3'
mm3_14	1st	5'-GACACGTGGCTGCAGAAGTA-3'	5'-GGGTCAGGATCAATGTCAGG-3'
	2nd	5'-GTGGCTGCAGAAGTATATAA-3'	5'-GGATCAATGTCAGGATCAAC-3'
mm3_15	1st	5'-CACAGCCCAGAGGACAAAGT-3'	5'-ATCCATGAGGATGCAGGTTC-3'
	2nd	5'-CCCAGAGGACAAAGTCTGCA-3'	5'-GAGGATGCAGGTTCCATCCC-3'
mm2_16	1st	5'-GGCCCCTCAGAACAGGGAAG-3'	5'-CCAGAGTTGAGAGCCTGGAG-3'
	2nd	5'-CTCAGAACAGGGAAGGATTA-3'	5'-GTTGAGAGCCTGGAGACCCA-3'
mm3_17	1st	5'-TTACTGCTGAGCCACAATGG-3'	5'-TTGGACTTTTGCATCTGCTG-3'
	2nd	5'-GCTGAGCCACAATGGGAACT-3'	5'-CTTTTGCATCTGCTGATACC-3'
mm2_18	1st	5'-CAAAGGATCCCGAGTCTTCA-3'	5'-CTGCCTGCATCCTTCTATGA-3'
	2nd	5'-GATCCCGAGTCTTCAAATTA-3'	5'-GCATCCTTCTATGATTATCC-3'
mm3_19	1st	5'-GTTGAGAGCCCACTGCAAAT-3'	5'-GGGTTTGCTGCCTGATAGAC-3'
	2nd	5'-GAGCCCACTGCAAATTCAAG-3'	5'-GCTGCCTGATAGACTGGAAT-3'
mm3_20	1st	5'-AGTTGCCCCCTAAGCAGTTT-3'	5'-AGGATGTGGTTGGCATGATT-3'
	2nd	5'-CCCCCTAAGCAGTTTATCTC-3'	5'-GGTTGGCATGATTACAAATA-3'
mm3_21	1st	5'-GGCACTCAGTGGCTTTTCTT-3'	5'-ACAGCTCACAGCAACACCAG-3'
	2nd	5'-CAGTGGCTTTTCTTATCTGA-3'	5'-CACAGCAACACCAGATCCTT-3'
mm3_22	1st	5'-CGTTAACCCACTGAGCAAGG-3'	5'-AGCACTTCATGGAAGGGAAA-3'
	2nd	5'-CCCACTGAGCAAGGGCAGGG-3'	5'-CATGGAAGGGAAAGGAAGAG-3'
mm3_23	1st	5'-TCTCCAGCTGGTGGTTTCTT-3'	5'-GCCAAGCACTTTGGAGGATA-3'
	2nd	5'-GCTGGTGGTTTCTTACAGGT-3'	5'-GCACTTTGGAGGATACTAAA-3'
mm3_24	1st	5'-ATCTGAGGTTGCTGCTCAGG-3'	5'-TGCTTTTCCCTCTCCTTTCA-3'
	2nd	5'-GGTTGCTGCTCAGGGCTCGG-3'	5'-CCCTCTCCTTTCAGCCCCAT-3'
mm3_25	1st	5'-CCAGGTAGGGACATTGGAAA-3'	5'-GTTGGTCAGCTCTTGGAAGC-3'
	2nd	5'-GGGACATTGGAAAAAAGGGA-3'	5'-CAGCTCTTGGAAGCTTTAGT-3'

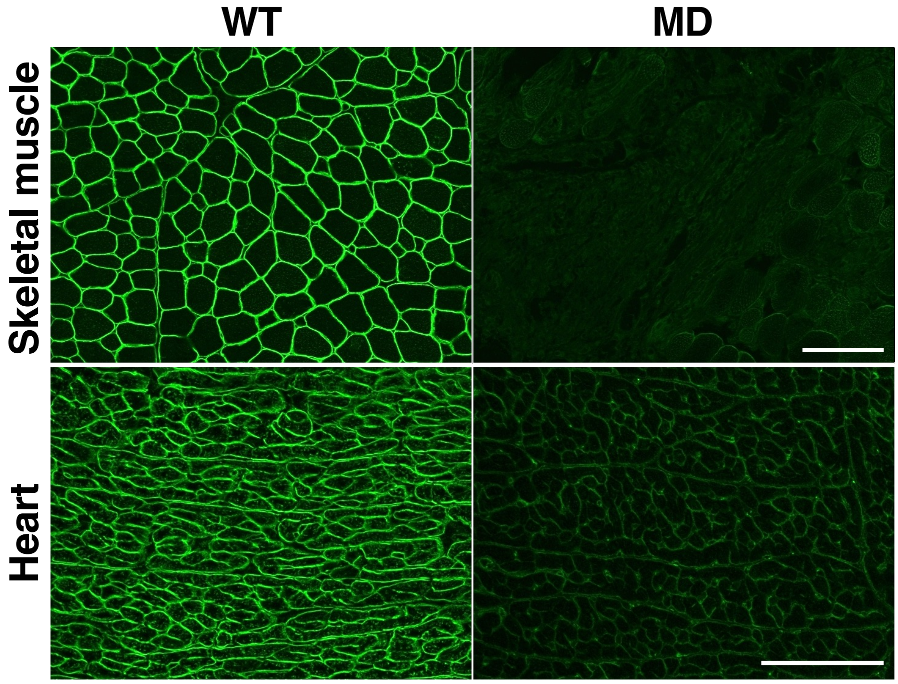

Supplementary Figure 1. Expression of dystrophin and utrophin in skeletal and cardiac muscles of *DMD*-edited microminipig.

Cryosections of tibialis anterior muscle (Skeletal muscle) and inter-ventricular septum (Heart) of two-month-old micriminipigs were subjected to immunofluorescence staining with an antibody against C-terminal of dystrophin (DYS-C in Fig. 2). Wild-type (WT) and *DMD*-edited microminipigs (MD). Bar, 100 μm.

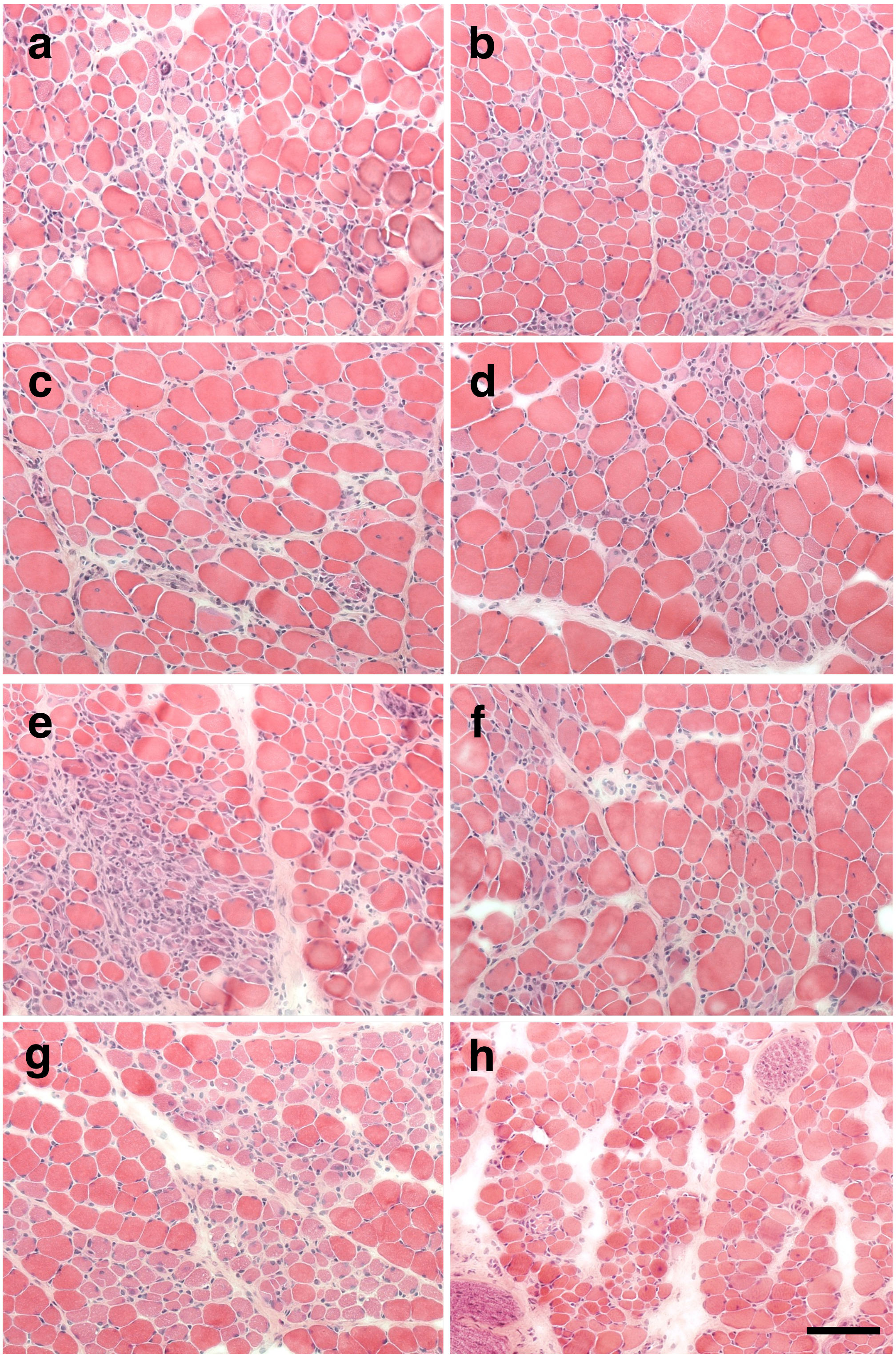

Supplementary Figure 2. Skeletal muscle pathology in *DMD*-edited microminipigs at two months of age.

H&E staining of eight different skeletal muscle cryosections using a two-month-old *DMD*-edited micromini pig. Micrographs show the gastrocnemius (a), rectus femoris (b), extensor digitorum longus (c), biceps brachii (d), longissimus dorsi (e), intercosal muscle (f), temporal muscle (g), and extraocular muscle (h). Bar, 100 μm.

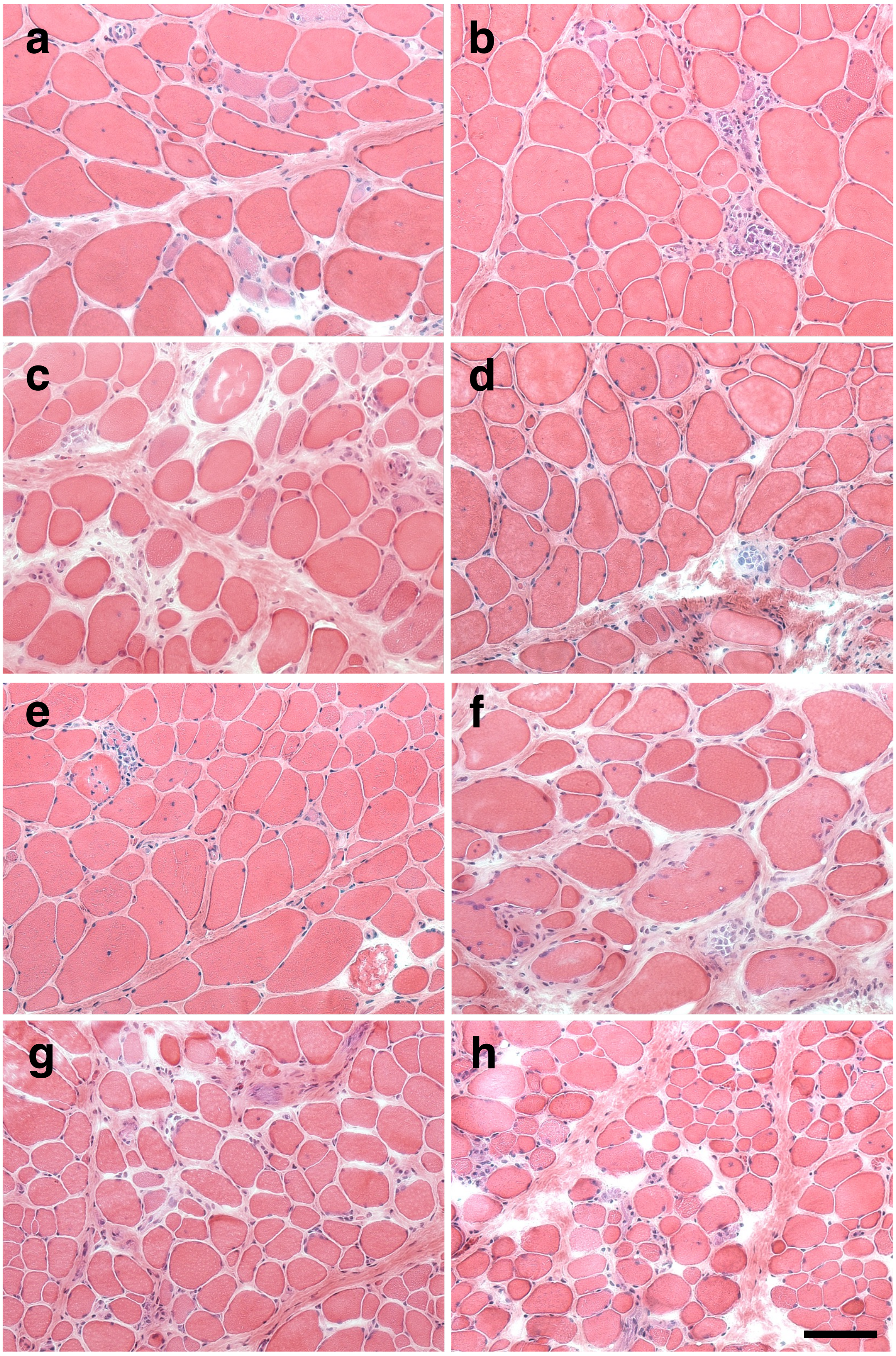

Supplementary Figure 3. Skeletal muscle pathology in *DMD*-edited microminipigs at 29 months of age.

H&E staining of eight different skeletal muscle cryosections using a 29-month-old *DMD*-edited microminipig. Muscles shown are the gastrocnemius (a), rectus femoris (b), extensor digitorum longus (c), biceps brachii (d), longissimus dorsi (e), intercosal muscle (f), temporal muscle (g), and extraocular muscle (h). Bar, 100 μm

Original Blots

Figure 2b

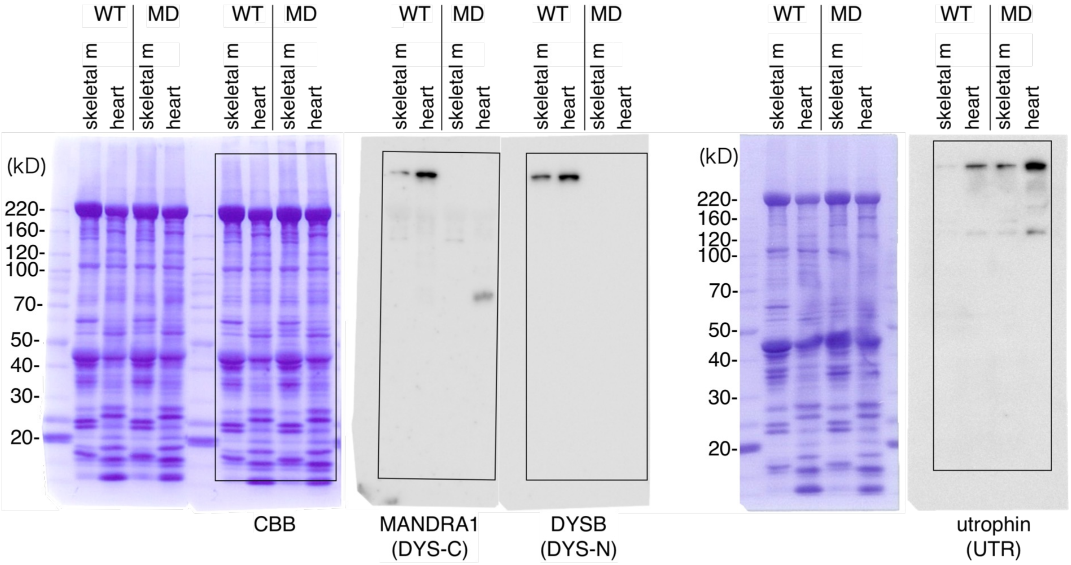

Figure 2c

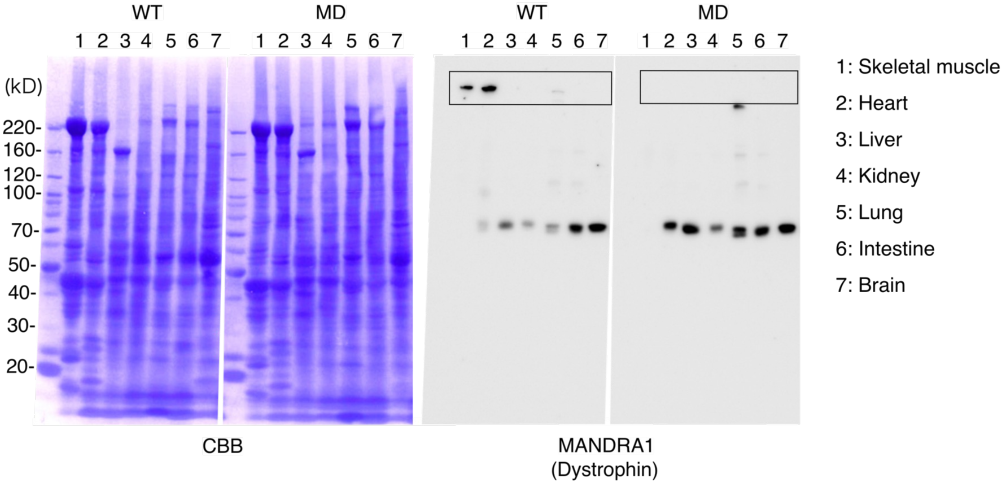

Figure 2c

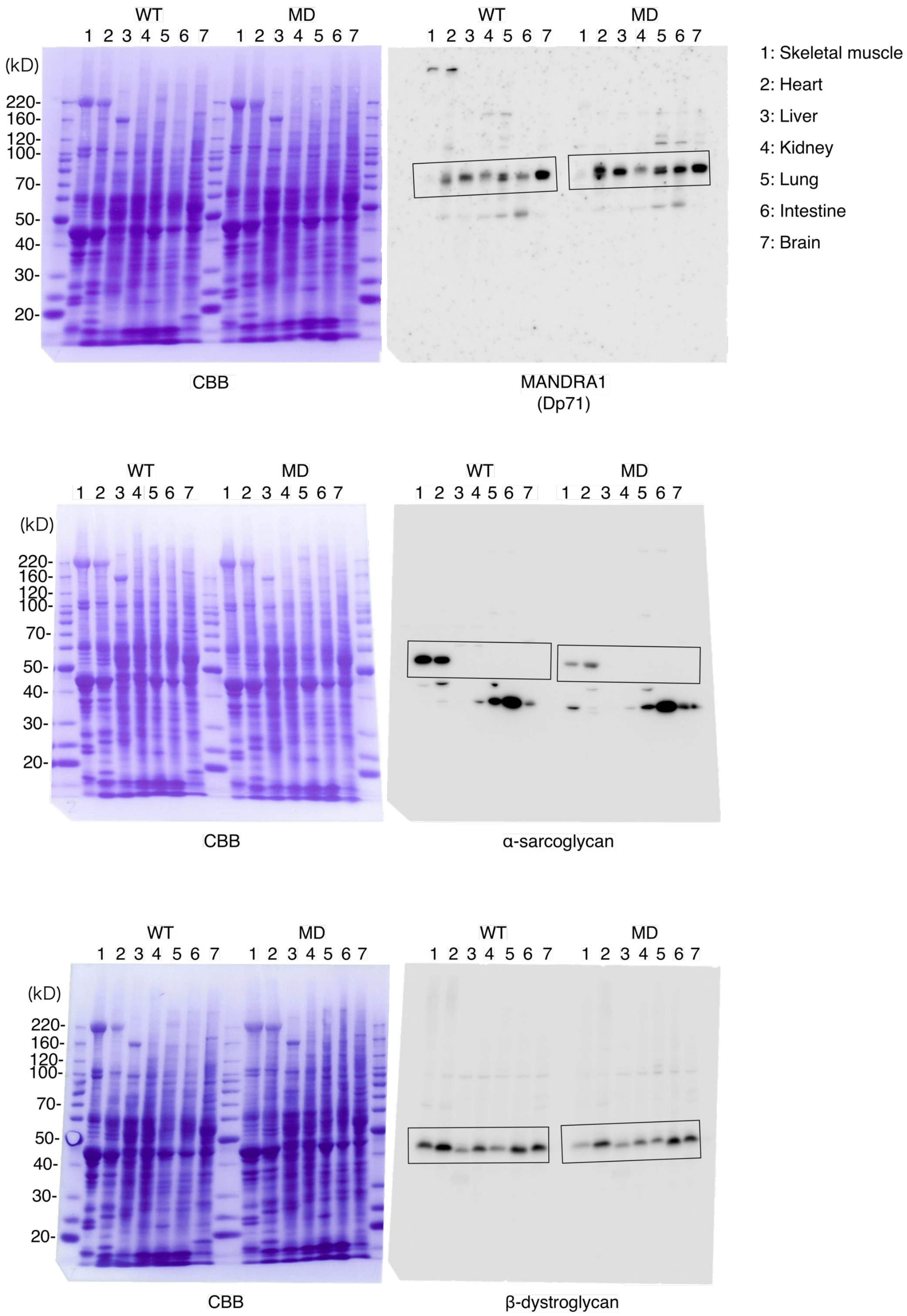

Supplementary Figure 4. Original uncropped blot and gel images of Figure 2b and 2c.
