# Supplementary material for: Severe cardiac and skeletal manifestations in DMD-edited microminipigs: an advanced surrogate for Duchenne muscular dystrophy
Source: Commun Biol. 2024 May 3;7:523. doi: 10.1038/s42003-024-06222-5 (PMC11068776; doi:10.1038/s42003-024-06222-5)
Supplement: Supplementary file 3 — Description of Additional Suppl Files [file 42003_2024_6222_MOESM3_ESM.pdf]

# Description of Additional Supplementary Files

**File name:** Supplementary Data 1

**Description:** The source data behind the graphs in Figure 4 of the paper.
