# Supplementary material for: Severe cardiac and skeletal manifestations in DMD-edited microminipigs: an advanced surrogate for Duchenne muscular dystrophy
Source: Commun Biol. 2024 May 3;7:523. doi: 10.1038/s42003-024-06222-5 (PMC11068776; doi:10.1038/s42003-024-06222-5)
Supplement: Supplementary file 5 — Reporting Summary [file 42003_2024_6222_MOESM5_ESM.pdf]

nature portfolio

Corresponding author(s): Yoshitsugu Aoki

Last updated by author(s): Mar 4, 2024

Reporting Summary

Nature Portfolio wishes to improve the reproducibility of the work that we publish. This form provides structure for consistency and transparency in reporting. For further information on Nature Portfolio policies, see our Editorial Policies and the Editorial Policy Checklist.

Statistics

For all statistical analyses, confirm that the following items are present in the figure legend, table legend, main text, or Methods section.

n/a	Confirmed	
☐	☒	The exact sample size (*n*) for each experimental group/condition, given as a discrete number and unit of measurement
☐	☒	A statement on whether measurements were taken from distinct samples or whether the same sample was measured repeatedly
☐	☒	The statistical test(s) used AND whether they are one- or two-sided<br>*Only common tests should be described solely by name; describe more complex techniques in the Methods section.*
☐	☒	A description of all covariates tested
☒	☐	A description of any assumptions or corrections, such as tests of normality and adjustment for multiple comparisons
☐	☒	A full description of the statistical parameters including central tendency (e.g. means) or other basic estimates (e.g. regression coefficient) AND variation (e.g. standard deviation) or associated estimates of uncertainty (e.g. confidence intervals)
☐	☒	For null hypothesis testing, the test statistic (e.g. *F*, *t*, *r*) with confidence intervals, effect sizes, degrees of freedom and *P* value noted<br>*Give P values as exact values whenever suitable.*
☒	☐	For Bayesian analysis, information on the choice of priors and Markov chain Monte Carlo settings
☒	☐	For hierarchical and complex designs, identification of the appropriate level for tests and full reporting of outcomes
☒	☐	Estimates of effect sizes (e.g. Cohen's *d*, Pearson's *r*), indicating how they were calculated

Our web collection on statistics for biologists contains articles on many of the points above.

Software and code

Policy information about availability of computer code

Data collection	No software was used.
Data analysis	ParmacoBasic ver.15.0.1

For manuscripts utilizing custom algorithms or software that are central to the research but not yet described in published literature, software must be made available to editors and reviewers. We strongly encourage code deposition in a community repository (e.g. GitHub). See the Nature Portfolio guidelines for submitting code & software for further information.

Data

Policy information about availability of data

All manuscripts must include a data availability statement. This statement should provide the following information, where applicable:

- Accession codes, unique identifiers, or web links for publicly available datasets
- A description of any restrictions on data availability
- For clinical datasets or third party data, please ensure that the statement adheres to our policy

Our study did not include the types of data listed above.

Research involving human participants, their data, or biological material

Policy information about studies with human participants or human data. See also policy information about sex, gender (identity/presentation), and sexual orientation and race, ethnicity and racism.

Reporting on sex and gender	*Use the terms sex (biological attribute) and gender (shaped by social and cultural circumstances) carefully in order to avoid confusing both terms. Indicate if findings apply to only one sex or gender; describe whether sex and gender were considered in study design; whether sex and/or gender was determined based on self-reporting or assigned and methods used. Provide in the source data disaggregated sex and gender data, where this information has been collected, and if consent has been obtained for sharing of individual-level data; provide overall numbers in this Reporting Summary. Please state if this information has not been collected. Report sex- and gender-based analyses where performed, justify reasons for lack of sex- and gender-based analysis.*
Reporting on race, ethnicity, or other socially relevant groupings	*Please specify the socially constructed or socially relevant categorization variable(s) used in your manuscript and explain why they were used. Please note that such variables should not be used as proxies for other socially constructed/relevant variables (for example, race or ethnicity should not be used as a proxy for socioeconomic status). Provide clear definitions of the relevant terms used, how they were provided (by the participants/respondents, the researchers, or third parties), and the method(s) used to classify people into the different categories (e.g. self-report, census or administrative data, social media data, etc.) Please provide details about how you controlled for confounding variables in your analyses.*
Population characteristics	*Describe the covariate-relevant population characteristics of the human research participants (e.g. age, genotypic information, past and current diagnosis and treatment categories). If you filled out the behavioural & social sciences study design questions and have nothing to add here, write "See above."*
Recruitment	*Describe how participants were recruited. Outline any potential self-selection bias or other biases that may be present and how these are likely to impact results.*
Ethics oversight	*Identify the organization(s) that approved the study protocol.*

Note that full information on the approval of the study protocol must also be provided in the manuscript.

Field-specific reporting

Please select the one below that is the best fit for your research. If you are not sure, read the appropriate sections before making your selection.

☒ Life sciences ☐ Behavioural & social sciences ☐ Ecological, evolutionary & environmental sciences

For a reference copy of the document with all sections, see nature.com/documents/nr-reporting-summary-flat.pdf

Life sciences study design

All studies must disclose on these points even when the disclosure is negative.

Sample size	The sample size was determined by excluding pigs that died suddenly from all disease model pigs obtained because the values of serum markers collected postmortem varied.
Data exclusions	No data were excluded from the analyses.
Replication	The reproducibility of the data cannot be verified at this time. This is because the data was obtained from all disease model pigs that have been obtained to date, so to confirm this reproducibility, it is necessary to use pigs that will be born in the future.
Randomization	The two groups to be compared were classified based on the presence or absence of genetic mutations, the test conditions were determined by age in months, and all individuals were tested. Therefore, it was not possible to arbitrarily select a sample and test it.
Blinding	Measurement of blood biomarkers was outsourced to an outside organization without informing the pigs' genotype.

Reporting for specific materials, systems and methods

We require information from authors about some types of materials, experimental systems and methods used in many studies. Here, indicate whether each material, system or method listed is relevant to your study. If you are not sure if a list item applies to your research, read the appropriate section before selecting a response.

Materials & experimental systems

n/a	Involved in the study
☐	☒ Antibodies
☒	☐ Eukaryotic cell lines
☒	☐ Palaeontology and archaeology
☐	☒ Animals and other organisms
☒	☐ Clinical data
☒	☐ Dual use research of concern
☒	☐ Plants

Methods

n/a	Involved in the study
☒	☐ ChIP-seq
☒	☐ Flow cytometry
☒	☐ MRI-based neuroimaging

Antibodies

Antibodies used

1. Anti-dystrophin antibody (DYSB), supplier; Novocastra Laboratories, product code; NCL-DYSB, clone; 34C5.
2. Anti-dystrophin antibody (MANDRA1), supplier; Merck/Sigma-Aldrich, catalog number; SAB4200763-100UL, clone; MANDRA1.
3. Anti-utrophin antibody (UT-2), supplier; self-made, clone; rabbit polyclonal.
4. Anti-beta-dystroglycan antibody (beta-DGs), supplier; self-made, clone; rabbit polyclonal.
5. Anti-alpha-sarcoglycan antibody (alpha-SG3), supplier; self-made, clone; rabbit polyclonal.
6. Peroxidase-conjugated anti-mouse antibody, supplier; Beckman Coulter, catalog number; PN IM0817, clone; polyclonal
7. Peroxidase-conjugated anti-rabbit antibody, supplier; Merck/Sigma-Aldrich, catalog number; MFCD00162788, clone; polyclonal
8. Alexa Fluor 488-conjugated anti-mouse antibody, supplier; Thermo Fisher Scientific, catalog number; A-11001, clone; polyclonal
9. Alexa Fluor 488-conjugated anti-rabbit antibody, supplier; Thermo Fisher Scientific, catalog number; A-11008, clone; polyclonal

Validation

1. Information about anti-dystrophin antibodies (DYSB), i.e. species, application, etc., can be found in the manufacturer's data sheet (file:///Users/admin/Downloads/dysb%20(3).pdf). Moreover, previous studies reported that dystrophin protein was successfully identified using this antibody.
2. Information about antibody (MANDRA1) is described in the manufacture's data sheet (https://www.sigmaaldrich.com/deepweb/assets/sigmaaldrich/product/documents/111/050/d8043dat.pdf). Moreover, many previous reports used this antibody to identify dystrophin.

3, 4, 5. Self-made anti-utrophin antibody (UT-2), anti-β-dystoglycan antibody (β-DG), and anti-α-sarcoglycan antibody (α-SG3) were reported about 20 years ago and have been used in several studies since then. The reactivity of these antibodies is shown in reference 36 of our manuscript.

Animals and other research organisms

Policy information about studies involving animals; ARRIVE guidelines recommended for reporting animal research, and Sex and Gender in Research

Laboratory animals

Animal species; pig, strain; microminipig, age; from 2 to 29 months old.

Wild animals

Our study did not involve wild animals.

Reporting on sex

Our study basically used mail pigs because Duchenne muscular dystrophy is X-linked recessive disease. However, due to the small sample size, one female was added to the control wild-type pigs. We do not specifically mention this female in the manuscript.

Field-collected samples

Our study did not involve samples collected from the field.

Ethics oversight

This study was approved by Animal Care and Use Committees of Shizuoka Prefectural Research Institute of Animal Industry, Swine and Poultry Research Center, and National Institute of Neuroscience.

Note that full information on the approval of the study protocol must also be provided in the manuscript.

Plants

Seed stocks

Report on the source of all seed stocks or other plant material used. If applicable, state the seed stock centre and catalogue number. If plant specimens were collected from the field, describe the collection location, date and sampling procedures.

Novel plant genotypes

Describe the methods by which all novel plant genotypes were produced. This includes those generated by transgenic approaches, gene editing, chemical/radiation-based mutagenesis and hybridization. For transgenic lines, describe the transformation method, the number of independent lines analyzed and the generation upon which experiments were performed. For gene-edited lines, describe the editor used, the endogenous sequence targeted for editing, the targeting guide RNA sequence (if applicable) and how the editor was applied.

Authentication

Describe any authentication procedures for each seed stock used or novel genotype generated. Describe any experiments used to assess the effect of a mutation and, where applicable, how potential secondary effects (e.g. second site T-DNA insertions, mosiacism, off-target gene editing) were examined.
